# The Gastropack Access System as a Model to Access Gastroenterology Services for Gastroscopy Appropriateness in Patients with Upper Gastrointestinal Symptoms: A Comparison with the Open Access System

**DOI:** 10.3390/jcm12093343

**Published:** 2023-05-08

**Authors:** Liza Ceroni, Francesca Lodato, Paolo Tubertini, Giovanni Marasco, Alessia Gazzola, Maurizio Biselli, Cristiano Fabbri, Federica Buonfiglioli, Francesco Ferrara, Ramona Schiumerini, Andrea Fabbri, Alessandra Tassoni, Carlo Descovich, Sandra Mondini, Cesare Tosetti, Valerio Veduti, Mario De Negri, Alessandro Fini, Stefano Guicciardi, Massimo Romanelli, Giuseppe Giovanni Navarra, Giovanni Barbara, Vincenzo Cennamo, on behalf of Gastropack System Study Group

**Affiliations:** 1 Department of Gastroenterology and Interventional Endoscopy, AUSL Bologna Bellaria, Maggiore Hospital Bologna, 40133 Bologna, Italy; 2 Process Reengineering, AUSL Bologna, 40124 Bologna, Italy; 3 Enterprise Information Systems for Integrated Care and Research Data Management, IRCCS Azienda Ospedaliero-Universitaria di Bologna, 40138 Bologna, Italy; 4 IRCCS Azienda Ospedaliero, Universitaria di Bologna, 40126 Bologna, Italy; 5 Department of Medical and Surgical Sciences, University of Bologna, 40126 Bologna, Italy; 6 Unit of Semeiotics, Liver and Alcohol-Related Diseases, IRCCS Azienda Ospedaliero, Universitaria di Bologna, 40126 Bologna, Italy; 7 Program for Clinical Governance and Outpatients Care, AUSL Bologna, 40124 Bologna, Italy; 8 Department of Clinical Governance and Quality, AUSL Bologna, 40124 Bologna, Italy; 9 Department of Primary Care, Distretto Appennino Bolognese, AUSL Bologna, 40124 Bologna, Italy; 10 Medical Direction, AUSL Bologna, 40124 Bologna, Italy; 11 Department of Biomedical and Neuromotor Sciences, University of Bologna, 40126 Bologna, Italy; 12 E-Care Processes Unit, AUSL Bologna, 40124 Bologna, Italy; 13 General Surgery, Distretto Appennino Bolognese, AUSL Bologna, 40124 Bologna, Italy

**Keywords:** endoscopy, general practice, shared medicine, guidelines

## Abstract

Esophagogastroduodenoscopy (EGD) appropriateness in Open-Access System (OAS) is a relevant issue. The Gastropack Access System (GAS) is a new system to access gastroenterological services, based on the partnership between Gastroenterologists and GPs. This study aims to evaluate if GAS is superior to OAS in terms of EGDS appropriateness. Secondarily, we evaluated the diagnostic yield of EGDS according to ASGE guidelines. The GAS was developed in an area of Bologna where General Practitioners (GPs) could decide to directly prescribe EGDS through OAS or referring to GAS, where EGDS can be scheduled after contact between GPs and specialists sharing a patient’s clinical information. Between 2016 and 2019, 2179 cases (M:F = 861:1318, median age 61, IQR 47.72) were referred to GAS and 1467 patients (65%) had a prescription for EGDS; conversely, 874 EGDS were prescribed through OAS (M:F = 383:491; median age 58 yrs, IQR 45.68). Indication was appropriate in 92% in GAS (1312/1424) versus 71% in OAS (618/874), *p* < 0.001. The rate of clinically significant endoscopic findings (CSEF) was significantly higher in GAS (49% vs. 34.8%, *p* < 0.001). Adherence to ASGE guidelines was not related to CSEF; however, surveillance for pre-malignant conditions was independently related to CSEF. All neoplasm were observed in appropriate EGD. GAS is an innovative method showing extremely high rates of appropriateness. ASGE guidelines confirmed their validity for cancer detection, but their performance for the detection of other conditions needs to be refined.

## 1. Introduction

Open-access endoscopy (OAE) allows primary health physicians (general practitioners) to directly refer patients for a routine gastrointestinal endoscopic procedure without a previous consultation with a Gastroenterologist [1]. In parallel with the increasing demand, OAE has become increasingly popular in recent years [2]. The use of OAE may reflect efforts to decrease costs for unnecessary office-based consultations. From 2000 to 2008, according to a nine-year retrospective audit, there has been a more than a fivefold increase in the number of open-access upper endoscopic procedures [3]. Although OAE is becoming more and more common, it can be associated with certain drawbacks such as poorly informed patients and inappropriate referrals, which can lead to a waste of resources [4,5,6,7,8]. In particular, many authors have focused on the correlation between the characteristics of the prescribing doctor (specialism, healthcare setting) and the appropriateness, and the diagnostic yield of the procedure [7,9,10,11,12,13]. Regarding the relationship between the specialism of the referring physician and appropriateness of esophagogastroduodenoscopy (EGD) according the ASGE guidelines, an Italian multicentric prospective study on 6270 patients involving 44 OAE centers found that the rate of “non indicated” procedures requested by specialists was significantly higher than the rate of procedures requested by general practitioners (GPs) [9]. The main reasons for inappropriate referrals are the unfamiliarity with guidelines, misunderstanding about the aetiology of symptoms, and therapy failure [14]. Different strategies have been proposed to reduce referral errors between GPs and specialists. Guidelines and education programs targeting primary care providers generally serve as the foundation for such interventions [15,16,17]. However, these interventions alone may be ineffective without opportunities for feedback and relationship building among medical professionals. In addition, although the recommendations highlight the comparison between GPs and specialists in the case of uncertain prescriptions, there is often a lack of structured and accessible communication among professionals [18]. Similarly, peer review or shared patient assessment may also reduce rates of unnecessary or delayed referrals and can be implemented via primary care triage clinics, multidisciplinary team-based diagnosis, and peer consultation groups [19]. From this point of view, the coordination between primary and secondary care is crucial to improve patient care and experience [20].

Several elements were identified as necessary for the integration of primary and secondary health care governance across a regional setting: shared planning, integrated information, communication technology and common clinical priorities [21]. Based on these considerations, we developed the Gastropack System (GAS), an integrated network between primary and secondary care professionals aimed at improving the effectiveness and efficiency of outpatient services at the primary–secondary care interface in the field of Gastroenterology.

This study aims to evaluate if GAS is superior to pre-existing Open Access System (OAS) in terms of appropriateness of upper endoscopic procedures.

A secondary endpoint is to evaluate the diagnostic yield of current international guidelines in terms of Clinically Significant Endoscopic Findings (CSEF) observation.

## 2. Methods

### 2.1. Study Design

All consecutive patients referred for upper GI symptoms in the Appennino District from May 2016 to May 2019 were included. All the patients provided written informed consent before enrolment. The study was approved by the institutional review board (Comitato Etico di Area Vasta Emilia Centro della Regione Emilia-Romagna, CE-AVEC registration number: 249/2019/OSS/AUSLBO) and complied with the provisions of the Good Clinical Practice guidelines and the Declaration of Helsinki.

For the GAS group, data were extracted from digital records prospectively filled by the GAS professionals, while the OAS population data were derived from GPs prescriptions and from the analysis of sanitary databases of patients. In both groups, data collection included age, sex, indication for EGD, and endoscopic and histological findings. Endoscopic diagnoses that were considered to be clinically significant or insignificant are reported in the corresponding section in Results. In the presence of several endoscopic diagnoses, the most severe one was used for statistical analysis.

Gastric preneoplastic lesions were defined as gastric intestinal metaplasia and dysplasia.

### 2.2. Study Groups

**Figure 1:**
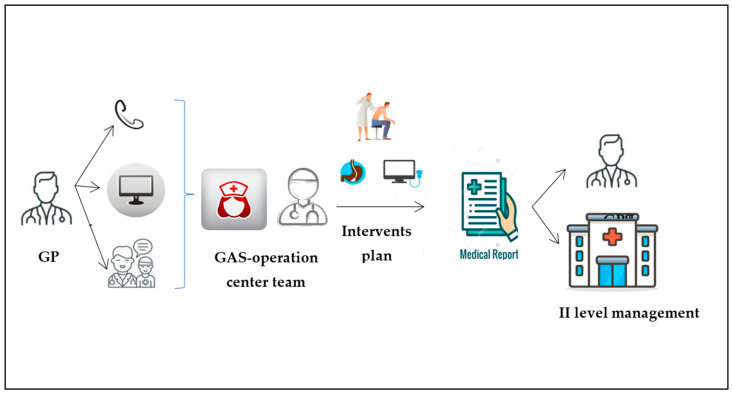
The GAS organization: GP and gastroenterologist share the patient’s clinical pathway; support staff organizes examinations in the predetermined time frame and merges them into the same day if possible. Once the pathway is concluded, the patient is referred back to the GP.

GAS has been developed since May 2016 in the Alto Reno Terme/Appennino District, a mountain area with 57,156 inhabitants of the Metropolitan City of Bologna: part of the Bologna Public Health Authority (AUSL Bologna). The population of this District is managed by 39 GPs. From the onset of the GAS, each GP was given the opportunity for each patient to freely join the new GAS or alternatively continue with the pre-existing OAS. The GAS derives from models of gastroenterological field experience including relocation of specialized facilities, and the shared planning and continuity of care by means of an information process. The system is based on a preliminary briefing between the referring GP and the Gastroenterologist. The GP could contact the GAS operation center in different ways: by telephone, a dedicated phone-line, a secure e-mail system, or by person. The GAS operation center team consists of a gastroenterologist and a trained nurse, who have access to a computerized trackable medical record in the RIS-PACS software called Polaris^®^. On this system the following data is recorded: patient’s personal data, main comorbidities, ongoing therapies (particularly, anticoagulant/antiplatelet therapy), presence of cardiac devices, allergies, gastroenterological history or endoscopic procedures. In addition to this data common to all reported patients, the discussion between the GP and Specialist is based on patient symptoms and results in a shared diagnostic work-up, which can consist of clinical visits, endoscopic procedures (EGD, colonoscopy) and abdominal and/or intestinal ultrasonography (US). The “gastroenterological pack” can include one or more consultations or exams with the assignment of a priority class [22], and leading to the final diagnosis. When all the procedures are performed, the Gastroenterologist closes the case with a diagnosis and indications for therapy, sending digitalized feedback to the GP, or in case of serious illness or complex chronic disease, referring the patient to the second level of management (Figure 1).

**Figure 2:**
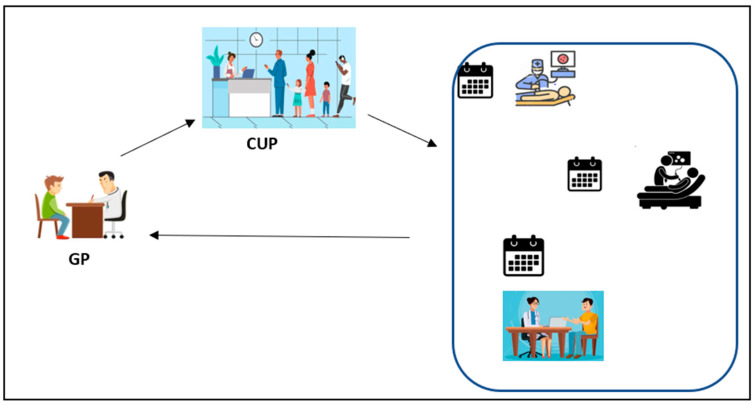
The OAS organization: The GP prescribes one or more services that are scheduled on different days and places according to the assigned priority and availability; after performing each test, the patient returns to the GP to show the result, and he can ask for further investigations.

Conversely, in the pre-existing OAS, the GP can directly prescribe a gastroenterological procedure (visit, endoscopy or US) without any previous contact with a specialist. The electronic prescription contains the clinical indication and the class of priority, as described above. The patient thus contacts a booking center (CUP, “Centro Unico Prenotazioni”), where the request is managed by non-medical personnel who arrange an appointment based on availability and the assigned priority with no evaluation in terms of appropriateness of indication and timing. Once procedures have been performed, the patient returns to the GP for evaluation and the GP may request further investigations if deemed necessary (Figure 2). The patient can be referred to a specialist only in particular clinical cases after the first visit.

Priority classes for procedures given by GPs or Gastroenterologists are the same for the two groups, in accordance with national guidelines (Urgent = 3 days, Short = 10 days; Deferrable = 60 days; Programmed = 180 days) [22]. The main difference is that in GAS, scheduling management is done by specialized personnel who decide, within a wide time frame, the relative priorities of individual requests. Based on the type of requests, the ambulatory program is built, which therefore is not rigid: examinations, endoscopies and ultrasounds are performed eventually in the same session because the starting point is the patient. Conversely, in the OAS, the patient must accept the first useful appointment, and if he or she has requests for different services, they must be performed on different days and possibly different locations because the patient must adapt to the prearranged organization. If, at the time of booking, the availability is finished, then the request is placed on a waiting list, and so, in these cases, the priority class is not respected. In GAS, the duration of the planning and execution phase of the pathway depends on the priority of the clinical case as defined at the moment of the interview with the GP. Conversely, the duration of the course in the OAS cannot be defined because it is the patient’s responsibility to report the results of prescribed services to the GP.

### 2.3. Statistical Analyses

**Figure 3:**
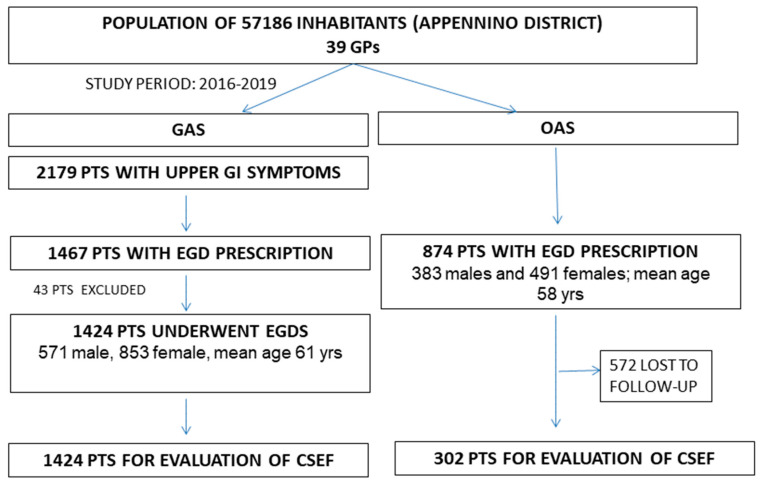
Flowchart of the study populations.

Qualitative variables were summarized using counts and percentages. Quantitative variables were expressed as mean ± standard deviation or median and interquartile range (IQR), as appropriate. Comparisons between groups were made by χ^2^-test or Fisher’s exact test for qualitative variables, and the Mann–Whitney test for quantitative variables. To ensure an unbiased comparison between the two groups, patients who refused to undergo EGD were excluded. In fact, while in the GAS group, consent denial was recorded, and in the OAS group, this information was untraceable. In addition, in the OAS group, endoscopic findings were available only in the minority of patients who underwent EGD in the Bologna AUSL area, whereas CSEF was performed only on a sub-group of patients with available data. A flowchart of study population is shown in Figure 3. Uni- and multivariate logistic regression analyses were performed to evaluate the association among the presence of CSEF and demographic parameters, the inclusion in GAS or OAS group, each ASGE indication and their appropriateness. Variables with a *p*-value < 0.10 were entered into the multivariate logistic model in order to identify independently significant variables. The Odds Ratio (OR) and 95% Confidence Intervals (95%CI) were calculated for each independent variable. All reported *p*-values are two-sided with *p* < 0.05 indicating statistical significance. All analyses were carried out using STATA SE 17.0 (Stata Corp., College Station, TX, USA) and SPSS version 23 (SPSS, Inc., Chicago, IL, USA).

## 3. Results

### 3.1. Study Populations

During the 3-year study (2016–2019) period in the Appennino District, 2179 cases (861 males and 1318 females, median age 61, IQR 47.72) were referred to GAS for upper GI symptoms. Among the district, 33/39 (84.6%) GPs, joined the GAS project. Among them, 1467 patients (67%) had a prescription for EGD and 712 (35%) did not. Three percent of patients (43 of 1467 patients) having an EGD prescription eventually refused it and were excluded from the analyses according to the study design; therefore, 1424 patients finally underwent EGD.

In the same period and from the same area, 874 patients had a prescription for EGD in the OAS (383 males and 491 females; median age 58 yrs, IQR 45.68). Of these, only one in three (302 pts) underwent EGD in Bologna, while no data about the endoscopic report were available for the remaining 572 patients. Demographic characteristics and clinical indication for EGD are reported in Table 1.

### 3.2. Appropriateness of EGD Indication

Main indications for endoscopy according to ASGE criteria [7,23] in the two groups are shown in Table 1. Indication to EGD was considered appropriate in 92% of cases in the GAS population versus 71% in the OAS group (1312/1424 vs. 618/874, *p* < 0.001) (Figure 3).

When comparing baseline characteristics, patients who had an inappropriate prescription for EGD were younger in both study groups (median age 50 vs. 62 yrs in GPS group, *p* < 0.001; 53 vs. 60 yrs in OAS group, *p* < 0.001). No gender difference was found.

Considering that EGD was inappropriately recommended more often in younger patients, we analyzed the appropriateness of indication according to age in both groups, as shown in Table 2 and Table 3. The cut-off of 45 years was selected coherently with the threshold adopted by the ASGE criteria for EGD indication [5]. In both age groups, GAS’s prescriptions were significantly more appropriate than OAS’s. With only a few exceptions, GAS showed better results in almost all indications, whereas the most alarming symptoms (i.e., GI bleeding) were equally recognized as appropriate indications for endoscopy in all age groups.

### 3.3. Clinically Significant Endoscopic Findings (CSEF)

**Figure 4:**
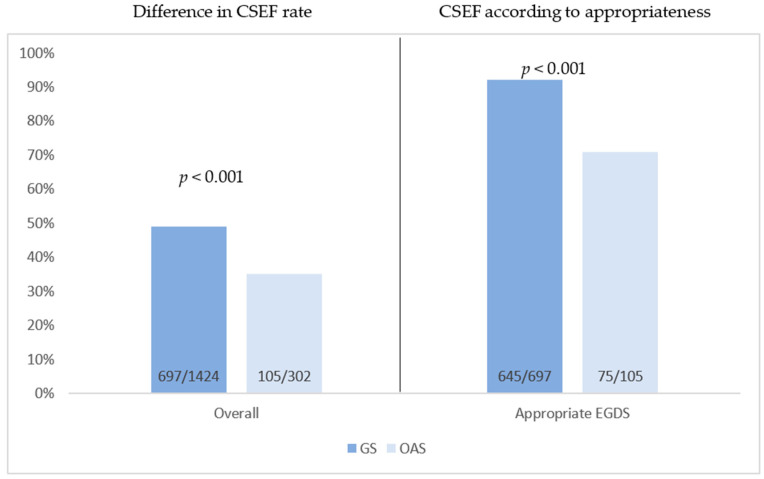
Difference in CSEF rate according to group (GAS vs. OAS) and appropriateness of EGDS.

According to the study design, CSEF were detected in a higher rate of patients in the GAS group when compared to the OAS group, namely, in 697 of 1424 EGD (49%) of the GAS group and 105 of 302 EGD (34.8%) of the OAS group, *p* < 0.001 (Figure 4).

In patients with CSEF, there was a statistically significant difference in the rate of appropriate EGD in the GAS group (92.5%) compared to the OAS group (71.4%), *p* < 0.001. According to Table 4, the most frequent endoscopic findings in the GAS group were erosive gastro-duodenitis and preneoplastic lesions, including gastric or duodenal dysplasia and metaplasia, while in the OAS group were erosive esophagitis, gastro-duodenitis and Barrett’s esophagus.

There was a difference in the rate of diagnosis of specific CSEF according to the study group. The rate of erosive esophagitis found in the OAS group was significantly higher compared to the GAS group (58% in OAS vs. 26% in GAS, *p* < 0.001), while the rate of preneoplastic lesions diagnosis and celiac disease was significantly higher in the GAS group compared to the OAS group (31% in GAS vs. 1.9% in OAS, *p* < 0.001).

Surprisingly, in the GAS group, CSEF rate did not differ significantly when EGD was performed with appropriate or inappropriate indication [645/1312 (49%) vs. 52/112 (46%), respectively; *p* = 0.516], similar to the OAS group when EGD was performed with appropriate or inappropriate indication [75/218 (34.4%) vs. 30/84 (35.7%), respectively, *p* = 0.830]. A detailed list of the endoscopic findings reported in each group is shown in Table 4.

At univariate analysis for the assessment of factors associated with CSEF and EGD appropriateness (OR 1.015, 95% CI 0.770–1.340, *p* = 0.914), and whether EGD was performed in GAS or OAS (OR 0.978, 95%CI 0.754–1.250, *p* = 0.818) were not associated with CSEF. On the other hand, patient’s age (OR 1.017, 95%CI 1.012–1.022, *p* < 0.001), female gender (OR 0.720, 95%CI 0.609–0.852, *p* < 0.001) and certain ASGE criteria [surveillance for premalignant conditions (OR 4.449, 95%CI 2.396–8.259, *p* < 0.001) and symptoms considered functional (OR 0.121, 95%CI 0.0679–0.215, *p* < 0.001)] were associated with CSEF. Factors that finally resisted at multivariate analysis were patient’s age (OR 1.009, 95%CI 1.004–1.015, *p* = 0.002), female gender (OR 0.738, 95%CI 0.616–0.885, *p* = 0.001) and certain ASGE criteria [surveillance for premalignant conditions (OR 3.980, 95%CI 2.136–7.414, *p* < 0.001) and symptoms considered functional (OR 0.130, 95%CI 0.072–0.233, *p* < 0.001)].

## 4. Discussion

In the last 30 years, the number of OAE procedures increased considerably in response to the increasing prescriptions mainly from GPs, according to a simple “supply and demand” mechanism. Nevertheless, this approach ignored an analysis of the real need and appropriateness of GI endoscopy, which is crucial when considering possible exposure to the unnecessary risk of an invasive procedure [1,2,3,7,24,25,26].

Commonly accepted guidelines specified indications for the appropriate endoscopy with the main goal not to miss serious diseases, such as cancer. In contrast, several studies demonstrated how the application of such guidelines on a large scale does not guarantee compliance with the appropriateness criteria [27,28,29].

Based on these considerations, we developed the GAS: a model of an integrated network between primary and secondary care professionals aimed at improving the effectiveness and efficiency of outpatient services in the field of Gastroenterology. This study is focused on the performance of the GAS compared to OAS, in the context of appropriateness of prescription for upper GI endoscopy.

Firstly, when applied to clinical practice, our model demonstrated an impressive 92% of EGD appropriateness according to ASGE criteria, which is the highest rate ever reported in literature [6,7,8,9,10]. In our study, GPs overprescribed EGD in 29% of the cases, coherently with data previously reported by Hassan et al. [9]. However, the same authors reported a rate of appropriateness of 87% for prescriptions given by specialists, while previous studies reported percentages not exceeding 79% [2,7,11,23,30] which are still significantly lower to those of GAS. Hence, it can be speculated that the main strength of our method lay on shared medicine, where GP and consultant contributions do not simply add one to another, but exponentially increase performance quality probably because a shared decision is stronger, and mutual monitoring among professionals leads to more virtuous prescriptive behavior. Inappropriate prescriptions were reported more frequently in younger patients (≤45 years) in both groups, and in this group of patients, both in GAS than in OAS, about 80% of inappropriate prescriptions were for functional symptoms, which also resulted in a protective factor for CSEF at multivariate analysis, as discussed later. Again, the GAS was superior to the OAS in terms of EGD appropriateness according to age populations in almost all benign indications considered (Table 2), while the most alarming symptoms were correctly identified in both GAS and OAS.

Adherence to appropriateness criteria is very important. Firstly, it means avoiding unnecessary invasive services for benign conditions. Secondly, we need to consider that we are in the context of a system with limited resources; thus, wastage impacts on the whole community. If a system has a high rate of inappropriateness, then availability is a burden for all individuals in the system, because the risk of saturation of places is higher and more difficult to ensure that services are performed in the required time. This is what happens in the OAS where the patient is placed on a waiting list when there is no availability for the requested exam. Conversely, in GAS, the scheduling is arranged by trained staff on a case-by-case basis to always adhere to the priority agreed upon by the physicians.

Secondly, our study aims to evaluate if high adherence to ASGE guidelines translates into a higher detection of endoscopic findings. In general, CSEF were significantly more frequent in GAS compared to the OAS group. With regard to appropriateness, we observed that the rate of CSEF was significantly higher in patients with an appropriate indication to EGD in the GAS group compared to the OAS group.

Coherently, with previous data [9,11], CSEF was reported in almost half of the patients in the GAS group; this proportion was lower in the OAS group (697/1424, 49% in the GAS group vs. 105/302, 34.8% in the OAS group). Surprisingly, in our study CSEF rate did not differ significantly when EGD was performed with no appropriate indication: in GAS, CSEF were 645/1312, 49% in appropriate EGD and 52/112, 46% in inappropriate EGD, and similarly to the OAS group, CSEF were 75/218, 34.4% in appropriate EGD and 30/84, 35.7% in inappropriate EGD. In contrast, a recent systematic review by Zullo et al. [31]. on upper gastrointestinal endoscopy appropriateness found a significant difference in terms of CSEF occurrence when comparing appropriate and inappropriate procedures according to current guidelines (43.3% vs. 35.1%, respectively). Nevertheless, it must be noted that in our study, the rate of CSEF in GAS group, both in appropriate and inappropriate EGDs, is higher compared to the rate reported in the review by Zullo et al. (49% and 46% in our study compared to 43.3 and 35.1%). It can be hypothesized that our shared enrollment system added diagnostic power to cases non-classifiable as appropriate, which allowed the diagnosis of clinically relevant lesions that otherwise would have been missed. We believe that the knowledge of patients and their clinical features by the GP, combined with a more rigorous approach by the specialist, may have contributed to this result.

In spite of the significant proportion of CSEF in inappropriate EGD, it must be noted that all neoplastic lesions were observed when an endoscopy was performed according to ASGE criteria; thus, confirming their value for cancer detection. On the one hand, it can be argued that guidelines were conceived favoring sensibility over specificity as required for screening purposes, and on the other hand other, relevant diagnosis (i.e., celiac disease, Barrett’s esophagus and pre-malignant lesions) would have been missed if the same guidelines had been followed strictly. Moreover, adherence to ASGE guidelines failed to relate to CSEF detection at multivariate analysis. However, when assessing each ASGE indication separately, surveillance for pre-malignant conditions emerged as an independent variable correlated to CSEF observation, and thus, further confirming the importance of not missing these conditions. Regarding this issue, we observed a significant difference in the observation of gastric pre-neoplastic lesions among the two groups: 31% in GAS vs. 1.9 in OAS. In our center, there are no strict protocols for biopsy collection with the exception of a specific situation according to standard quality endoscopy behavior (for example, known or suspected Barrett esophagus or chronic atrophic gastritis, or the histological confirmation of celiac diseases). For the remaining situations, each gastroenterologist works according to their training and the knowledge of the guidelines. Certainly, a common attitude can be found among professionals working in the same hospital group. In addition, the GAS allows the endoscopist performing the EGD a complete picture of the patient’s problems, who has already been taken care of by the system (as described in Methods). This probably also explains the increased attention given to biopsy sampling and accounts for the important differences in the rates of pre-neoplastic lesions. Similarly, we report a higher rate of celiac disease diagnosis; again, this is probably because in GAS, EGD is often the conclusion of a diagnostic path and a duodenal biopsy represents the end of this process. This suggests that possibly, in an open system, some celiac diseases with non-typical symptoms undergo endoscopic procedures unnecessarily or that many celiac diseases escape diagnosis in non-standardized pathways.

Other factors related to significant endoscopic findings at EGD were male sex and patient’s age, coherently with ASGE criteria [5], while functional symptoms resulted in a protective factor for CSEF at EGD, confirming that in the presence of such symptoms, EGD should not be indicated [26].

Overall, these results suggest that while ASGE guidelines are effective in the detection of cancer, future refinement should aim to tailor recommendations according to age risk and to improve the detection of other relevant GI conditions.

Our study has several strengths, such as the proposal of an innovative model for Gastroenterology outpatient care with outstanding performance results and the relatively large and homogeneous single-center cohort, followed prospectively under an established protocol to ensure a solid analysis of endoscopic findings in relation to guidelines adherence.

The main limitation of our study is the control group. In fact, while the data of the GAS group were collected prospectively, those of the OAS group were retrospectively collected and analyzed, theoretically impairing the comparison to GAS. In addition, data about CSEF in the OAS group were available only in 34.5% (302/874), because in this group, most procedures were untraceable since they were not performed by our Service considering the vast offer both in the public and private sector in the Bologna area. However, the scattering of OAS data is itself informative. Indeed, in OAS the GP prescribes diagnostic procedure for whomever organizes appointments by himself, with the risk of performing unnecessary procedures with inadequate timing and sequence, and possibly in unqualified centers. In contrast, in the GAS, no data or time are lost because the patient is accompanied along a protected path with a rational and well-defined program. Thus, the GAS should not be considered a simple tele-consult, but a global management of the patient to respond to his need for care.

Another missing data is relative to the group of patients who did not have EGD. In GAS, the patient is taken in charge and followed up until diagnosis, but we do not know if after the conclusion of the pathway, the same patient performed EGD with different modalities, and therefore, something was missed during the study. This problem is even greater for the OAS group, in which the analysis was done retrospectively by collecting data from the health registry, so in this group we cannot know whether in some cases, EGD should have been prescribed and if this led to delayed or missed diagnosis of some relevant diseases.

The GAS demonstrated the highest quality performance ever reported. It must be underlined that the purpose of this study is to propose a valid alternative method in response to the emerging issue of over-demand. In recent years to address this issue, the importance of a shared interdisciplinary approach has been underlined in all fields of medicine even though it almost exclusively involves specialists of different disciplines (i.e., in multidisciplinary tumor boards), while GPs are usually taken apart from clinical management. Indeed the OAE reference documents [1] theoretically recommend a consultation between GP and specialists in cases of uncertain prescription. However, GPs are often discouraged to contact specialists due to the lack of a direct communication channel, often resulting in an overprescription in order to avoid medico-legal issues. In previous experiences, a ‘censored’ OAE approach was analyzed, selecting patients for endoscopy after a pre-endoscopic filter evaluation by the gastroenterologist [32]. However, this strategy proved inefficient in reducing endoscopic procedures and proved uselessly time-consuming. Moreover, this approach implies a subordinate relationship between examination prescriber and performer, in contrast to the same-level partnership warranted by the GAS, which also demonstrated to be advantageous in terms of appropriateness.

In conclusion, the GAS is an innovative and efficient method based on the partnership between Gastroenterologist and GPs, ensuring every patient has a well-structured and appropriate diagnostic plan. This system demonstrates high quality standards in terms of the appropriateness of EGD prescription for upper GI symptoms, which represent a major reason for specialist referral. Although its value in other settings (i.e., colonoscopy, abdominal US) and its cost-effectiveness and feasibility in larger scale scenarios deserve further investigation, the GAS undoubtedly represents a promising and reasonable alternative to the traditional OAS. In addition, ASGE guidelines confirms their validity for cancer detection, but their performance for the detection of other relevant conditions needs to be refined.

## Figures and Tables

**Table 1:** Comparison between GAS and OAS based on demographic characteristic and main indication to EGD according to ASGE criteria in the OAS and GAS groups.

	GAS Group (1424 Cases)	OAS Group (874 Cases)	*p*
***Demographic characteristic of patients***			
Age (median [IQR]), years	61 [49–72]	58 [45–68]	<0.01
Gender (n, %), M/F	571 (40)/853 (60)	383 (44)/491 (56)	0.043
***Appropriate indication***	1312 (92%)	618 (71%)	<0.001
Upper pesistent symptoms	75 (5.7)	97 (15.7)	<0.001
Upper symptoms suggesting organic disease or in pts > 45 yrs	445 (33.9)	154 (24.9)	<0.001
Esophageal reflux symptoms persistent or recurrent	451 (34.4)	204 (33.0)	Ns
Portal hypertension evaluation	7 (0.5)	10 (1.6)	0.02
Active or recent bleeding	51 (3.9)	27 (4.4)	Ns
Chronic blood loss or iron deficiency anemia	99 (7.5)	21 (3.4)	<0.001
Tissue sampling	5 (0.4)	23 (3.7)	<0.001
Dysphagia	68 (7.2)	19 (3.1)	0.045
Persistent vomiting	47 (3.6)	16 (2.6)	Ns
Surveillance of premalignant conditions	64 (4.9)	33 (5.3)	Ns
***Not appropriate indication***	112 (8%)	256 (29%)	<0.001
Symptoms considered functional	48 (33.0)	127 (49.6)	<0.001
Surveillance of healed benign lesions	6 (5.4)	40 (15.6)	0.006

**Table 2:** Main indication to EGD in OAS and GAS groups in pts < 45 years (modified from ref. [5]).

	GAS *n* (%) 252 pts	OAS *n* (%) 220 pts	*p*
***Appropriate indication***	205 (81)	125 (57)	<0.001
Upper persistent symptoms despite an appropriate trial of therapy	9 (4.4)	31 (24.8)	<0.001
Upper symptoms suggesting organic disease or pts > 45 years	86 (42)	19 (15.2)	<0.001
Esophageal reflux symptom persistent or recurrent	57 (28)	35 (28)	Ns
Portal hypertension evaluation	-	-	
Active or recent bleeding	8 (3.9)	8 (6.4)	Ns
Chronic blood loss or iron deficiency anemia	20 (9.8)	1 (0.8)	<0.001
Sampling of tissue	-	7 (5.6)	0.05
Dysphagia	12 (5.9)	4 (3.2)	Ns
Persistent vomiting	12 (5.9)	7 (5.6)	Ns
Surveillance of premalignant condition *	1 (0.5)	6 (4.8)	0.04
***Not appropriate indication***	47 (19)	95 (43)	<0.001
Symptoms considered functional	38 (80)	77 (81.1)	Ns
Surveillance of healed benign lesions	2 (4.3)	10 (18.6)	0.01

* Pre-malignant condition, as defined in methods, were considered gastric metaplasia or dysplasia, chronic atrophic gastritis, Barrett diseases.

**Table 3:** Main indication to EGD in OAS and GAS groups in pts > 45 years (modified from ref. [5]).

	GAS *n* (%) 1172 pts	OAS *n* (%) 654 pts	*p*
***Appropriate indication***	*n* = 1107 (95)	*n* = 493 (75)	<0.001
Upper pesistent symptoms despite an appropriate trial of therapy	66 (6%)	66 (13.4)	<0.001
Upper symptoms suggesting organic disease or pts > 45 yrs	359 (32.4)	135 (27.4)	0.04
Esophageal reflux symptom persistent or recurrent	394 (35.6)	169 (34.3)	Ns
Portal hypertension evaluation	7 (0.6)	10 (2)	0.01
Active or recent bleeding	43 (3.9)	19 (3.9)	Ns
Chronic blood loss or iron deficiency anemia	79 (7.1)	20 (4.1)	0.01
Sampling of tissue	5 (0.5)	16 (3.2)	<0.001
Dysphagia	56 (5.1)	15 (3)	Ns
Persistent vomiting	35 (3.2)	9 (1.8)	Ns
Surveillance of premalignant condition *	63 (5.7)	27 (5.5)	Ns
***Not appropriate indication***	65 (5)	*n* = 161 (25)	<0.001
Symptoms considered functional	10 (15)	50 (31.1)	0.001
Surveillance of healed benign lesions	4 (6.2)	30 (18.6)	0.011

* Pre-malignant condition, as defined in methods, were defined as gastric metaplasia or dysplasia, chronic atrophic gastritis, Barrett diseases.

**Table 4:** CSEF in GAS and OAS group.

	GAS Group	OAS Group	*p*	GAS Group			OAS Group		
CSEF	697/1424 (49%)	105/302 (34.8%)	<0.001	Appropriate Indication 645/697 (92.5%)	Inappropriate Indication 52/697 (7.5%)	*p*	Appropriate Indication 75/105 (71.4%)	Inappropriate Indication 30/105 (28.6%)	*p*
Erosive gastroduodenitis	143 (20%)	21 (20%)	Ns	134 (21%)	9 (17%)	Ns	12	9	Ns
Erosive esophagitis	184 (26%)	61 (58.1%)	<0.001	170 (26%)	14 (26%)	Ns	41	20	Ns
Gastric ulcer	20 (2%)	2 (1.9%)	Ns	20 (3%)	0	Ns	2	0	Ns
Duodenal ulcer	24 (3%)	1 (1)	Ns	22 (3%)	2 (4%)	Ns	1	0	Ns
Bleeding lesions	2 (0.2%)	1 (1)	Ns	2 (3%)	0	Ns	0	1	Ns
Gastric preneoplastic lesions	217 (31%)	2 (1.9)	<0.001	198 (30%)	19 (36%)	Ns	2	0	Ns
Portal hypertension	7 (1%)	2 (1,9)	Ns	7 (1%)	0	Ns	2	0	Ns
Celiac disease	26 (4%)	1 (1)	0.005	24 (4%)	2 (4%)	Ns	1	0	Ns
Barrett’s esophagus	25 (4%)	9 (8.3)	0.032	22 (4%)	3 (4%)	Ns	9	0	0.057
Submucosal lesions	17 (2%)	0	Ns	15 (2%)	2 (4%)	Ns	0	0	-
Esophageal stenosis	5 (0.7%)	3 * (2.9)	0.075	4 (6%)	1 (2%)	Ns	3	0	Ns
Benign duodenal stenosis	3 (0.4%)	0	Ns	3 (0.4%)	0	Ns	0	0	-
Esophageal cancer	7 (1%)	1 (1)	Ns	7 (1%)	0	Ns	1	0	Ns
Gastric cancer	17 (2%)	1 (1)	Ns	17 (3%)	0	Ns	1	0	Ns

* Including 1 Zenker diverticula and 1 Bolus episode.

## Data Availability

Data is unavailable due to privacy or ethical restrictions.

## Appendix A. Gastropack System Study Group

Alberani Angela ^1^, Polifemo Anna Maria ^1^, Trevisani Franco ^5,6^, Gramenzi Annagiulia ^5,6^, Migliano Maria Teresa ^5,6^, Tovoli Stefano ^9^, Lenzi Adelmo ^9^, Brini Loris ^9^, Giovannelli Carlo ^9^, Inì Angela ^9^, Insarda’ Michele ^9^, Mori Mara ^9^, Montanari Elena ^9^, Musolino Angela ^9^, Pierallini Roberto ^9^, Riccioni Filippo ^9^, Vivarelli Serena ^9^, Maltese Cristina ^9^, Matteini Paola ^9^, Guzzardella Antonio Gaetano ^9^, Caporale Paola ^9^, Cavazza Luana ^9^, Cobianchi Corrado ^9^, De Negri Mario ^9^, Fini Pieraugusto ^9^, Gardenghi Amleto ^9^, Gasparini Carlo ^9^, Grazia Giuseppe ^9^, Liberini Bruno ^9^, Montanaro Domenico ^9^, Tursellino Luigi Carlo ^9^, Bartolomei Paolo ^9^, Bartolomei Vincenzo ^9^, Brazzi Giampaolo ^9^, Galuppi Stefano ^9^, Nanni Pier Giacomo ^9^, Sambataro Maria Concetta ^9^, Trombetti Elisa ^9^, Nanni Ilaria ^9^, Lattuca Thomas ^9^, Matteini Paola ^9^, Galasso Francesco Saverio ^9^.

1 Department of Gastroenterology and Interventional Endoscopy, AUSL Bologna Bellaria, Maggiore Hospital Bologna, 40133 Bologna, Italy
5 Department of Medical and Surgical Sciences, University of Bologna, 40126 Bologna, Italy
6 Unit of Semeiotics, Liver and Alcohol-related Diseases, IRCCS Azienda Ospedaliero, Universitaria di Bologna, 40126 Bologna, Italy
9 Department of Primary Care, Distretto Appennino Bolognese, AUSL Bologna, 40124 Bologna, Italy
